# Evaluation of recurrence, mortality and treatment complications of oral squamous cell carcinoma in public health centers in Shiraz during 2010 to 2020

**DOI:** 10.1186/s12903-023-03071-2

**Published:** 2023-05-30

**Authors:** Fahimeh Rezazadeh, Azadeh Andisheh-Tadbir, Zahra Malek Mansouri, Bijan Khademi, Pourya Bayat, Hossein Sedarat, Amir Tabesh, Elham Tayebi Khorami

**Affiliations:** 1 Oral and dental disease research center, Oral and Maxillofacial medicine Department, School of Dentistry, Shiraz University of Medical Sciences, Shiraz, Iran; 2 Prevention of Oral and Dental Disease Research Center, Department of Oral & Maxillofacial Pathology, School of Dentistry, Shiraz University of Medical Sciences, Shiraz, Iran; 3 Student Research Committee, School of Dentistry, Shiraz University of Medical Sciences, Shiraz, Iran; 4 Shiraz University of Medical Sciences, Shiraz, Iran; 5 Student Research Committee, Jahrom University of Medical Sciences, Jahrom, Iran

**Keywords:** Squamous cell carcinoma, Recurrence, Mortality, Complications, Oral

## Abstract

**Introduction:**

Oral Squamous cell Carcinoma (OSCC) is the most common oral cancer and is treated with surgery, radiotherapy and chemotherapy. Various complications of treatment include xerostomia, mucositis, and trismus, which affect patients’ quality of life. The aim of this study is to evaluate the mortality, recurrence rate and prevalence of oral complications in treated patients.

**Method and materials:**

This cross-sectional study reviewed 326 cases of patients with OSCC who were referred to public health centers in Shiraz (Khalili Hospital and Dental School) from 2010 to 2020. All patients were contacted, and the survivors were called and examined by an oral physician. A medical record was created for them, including demographic information, location of the lesion, type of treatment, history of recurrence, metastasis and oral complications.

**Results:**

53.5% of patients were male and 46.5% were female. The mean age of patients was 58.68 years. Mortality and recurrence rate was respectively 49.8% and 17.8%. The most common location of the lesion was tongue (64%). Surgery was done for all patients. 97.4% of patients complained of xerostomia, 46.2% of mucositis and 44.3% of trismus.

**Conclusion:**

The most common complications of treatment are xerostomia, mucositis, and trismus, respectively. Frequent and regular follow-ups and supportive therapies reduce these complications and improve patients’ quality of life.

## Introduction

Oral cancer is a broad term that includes a variety of malignancies in the tissues of the mouth. Control and prognosis may vary in different types and stages of oral cancer, which always has a significant impact on the patient’s life. Cancer and its treatment are associated with complications that may negatively affect the quality of life immediately after cancer treatment and throughout the patient’s life, which is related to the time of diagnosis and duration of cancer treatment [1]. Oral complications may lead to disruption of cancer treatment, affecting prognosis and increasing the importance of health care. The broad range of oral problems that often arise following cancer treatment make it difficult to diagnose and treatment plan. For example, severe mouth and throat pain due to inflammation of the oral mucosa, oro-pharyngeal candidiasis, decreased salivary gland function, and xerostomia often leads to dysphagia and nutritional problems in cancer patients [2]. Therefore, oral cancer treatment complications severely affect the quality of life during the treatment and months or years later.

Squamous cell carcinoma (SCC) is the most common oral cavity malignancy that regularly occurs in the soft tissues of the mouth. SCC is usually seen as an ulcer or red and white lesion in the soft tissue of the mouth, especially in the lateral border of the tongue [3]. Treatment plans vary from surgery, radiotherapy, chemotherapy or a combination of them, which cause different complications for the patient [4]. There are many factors that influence the type of treatment. It seems that the most important factors are the stage of cancer and tumor size. The other factors include age, gender, location and pathological grade of the tumor, drinking, and smoking [5]. Risk factors affecting the complications of treatment comprise preoperative radiotherapy, existence of other medical conditions, length of operation, and nutritional status [6].

In a similar study, Yok Fui Wong examined 130 patients with HNSCC. Their survival rate was estimated at 34.4% over five years. Also, 41.4% of patients complained of treatment complications [4]. In another study, a 15-year follow-up examined the history of smoking in patients with SCC and concluded that smoking had little effect on the survival rate [7].

The complications of SCC treatment, mortality rate, recurrence and metastasis of this disease are essential and maintaining follow-up is necessary for patients after treatment. Also, there has been no similar study in this field, specifically dealing with oral complications of SCC and treatment in Iran, in recent years. This study examined the mortality, recurrence, and oral complications of OSCC after treatment in public centers in Shiraz from 2010 to 2020.

## Method and materials

In this cross-sectional study, 326 patients with histopathological diagnosis of OSCC between 2010 and 2020 that referred to public health centers in Shiraz (Khalili Hospital and Dental School) after treatment period, were investigated and the stage of the disease was determined by clinical aspects and radiographic pictures (CT scan particularly).

This study was performed after approval by the ethics committee of Shiraz Dental School with the ethics ID IR.SUMS.DENTAL.REC.1399.204.

Patients with oral SCC who were treated and signed consent forms for examination were included in the study. Patients with systemic diseases such as autoimmune, inflammatory, infectious, and other cancers and medications that cause xerostomia or a change in taste or oral ulcers (side effects similar to treated SCC) were excluded from this study. For all patients, the examination was performed with a dental mirror on the dental unit with sufficient light.

**Fig. 1:**
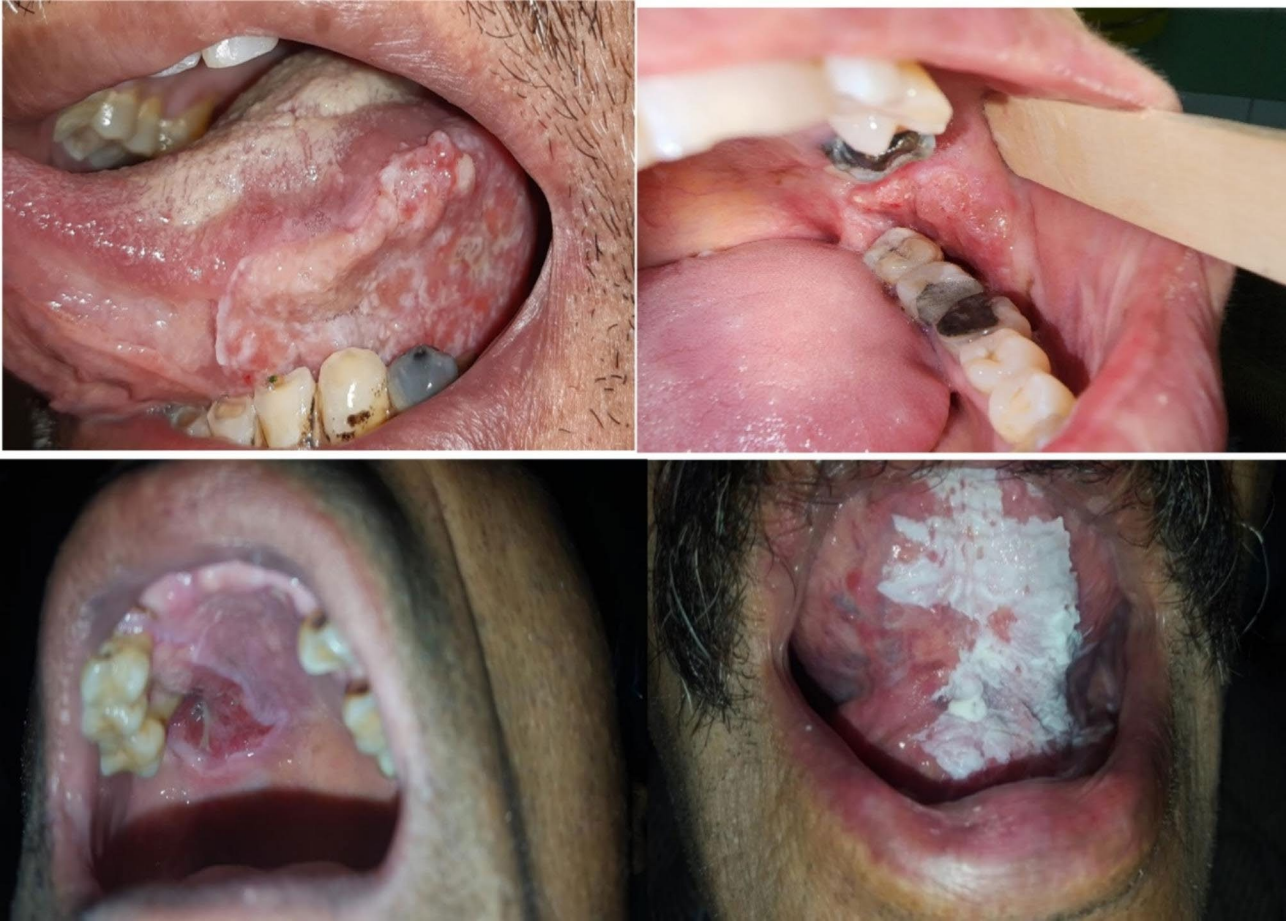
Clinical pictures from different types of oral Squamous Cell Carcinoma patients

Information data, including age, sex, financial status and level of education of the patients, were collected. Also, clinical pictures from different types of oral Squamous Cell Carcinoma patients were collected for better visualization of this disease condition. (Fig. 1)

The financial status of patients was divided into three groups based on the amount of income: 1- Poor: less than 2 million Tomans (Iran’s currency). 2- Average: 2 to 7 million Tomans. 3- Strong: more than 7 million Tomans. Patients’ level of education was also divided into three groups: 1- Undergraduate. 2- Diploma and post-diploma. 3. Bachelor’s degree and higher.

The location of the lesion, the type of treatment and the history of recurrence were also evaluated. Also, all oral complications, including xerostomia, inability to swallow, speak and eat, change in taste, fungal infections of the mouth, muscle fibrosis (inability to mouth opening), osteoradionecrosis and oral ulcers (mucositis), were evaluated. Xerostomia was assessed by tongue blade test, and patients were also asked about the feeling of dryness and difficulty swallowing. The presence of fungal infections was clinically evaluated by a dentist and approved by an oral medicine specialist. The history or presence of oral ulcers (mucositis) and the occurrence of osteoradionecrosis were assessed by a questionnaire, oral examination, and radiography if necessary.

To assess other oral complications, a questionnaire was prepared in which the patient reported the presence or absence of problems in speaking, swallowing and eating or inability to mouth opening (muscle fibrosis) and changes in the sense of taste.

Restrictions on mouth opening were assessed by measuring the maximum mouth opening with a Colimeter. To evaluate the change in the sense of taste, the patient gave one score for his sense of taste 1, 2 and 3, with a score of 1 meaning no sense of taste (lack of taste perception) and a score of 2 meaning a slight change of taste (perception of only some tastes). And a score of 3 meaning a sense of normal taste.

Finally, the data were entered into SPSS software version 23. Chi-square and t-test, ANOVA and Fisher exact test were used to analyze the data. Significance level (α) was considered 0.05.

## Results

In this study, 326 patients with SCC were investigated. 53.5% of the patients were male, and 46.5% were female. The mean age of the patients was 58.68±2 years. 24.3% of people had poor financial status and, 68.2% of people had moderate financial status and 7.4% of people with good financial status. 62% had undergraduate education, 31.2% had a diploma and postgraduate education, and 6.8% had a bachelor’s or a higher degree (Table 1).

**Table 1:** Demographic information of patients

Demographic information	Percent
Gender	Male 53.5% female 46.5%
Age	58.68
Financial status	Low 24.3% moderate 68% high 7.4%
Education	Undergraduate 62% diploma 31.2% bachelor 6.8%
Alcohol drinking	20.3%
smoking	34.5%

It was reported in 34.5% of smokers and 20.3% of alcohol users. All patients underwent surgery, and 52% of surgeries were performed by an oral and maxillofacial surgeon. 97.8% of patients underwent neck dissection surgery. The most common site of the lesion was the tongue (64%), and the second common site was the buccal mucosa (15.1%). (Table 2)

**Table 2:** Location of lesion

Location of lesion	Percent
Tongue	64%
Floor of the mouth	15.1%
Buccal mucosa	5.8%
Palate	3.1%
Alveolar process	2.8%
Salivary glands	1.2%
Body of mandible	0.3%
lips	0.3%

The mortality rate of SCC in this study was 49.8%, and 17.8% of patients reported recurrence after treatment (which was confirmed by histopathology).

The most adjunctive treatment was radiotherapy. 97.4% of the patients underwent radiotherapy, and 17.5% underwent chemotherapy in addition to radiotherapy (Table 3).

 .

**Table 3:** Type of treatment

Type of treatment	Percent
Surgery	100%
Surgery-radiotherapy	97.4%
Surgery-chemotherapy	17%
Surgery-radiotherapy-chemotherapy	31.7%

97.4% of patients complained of xerostomia, which was clinically confirmed by tongue blade test.

46.2% of patients reported oral mucositis( 91% after radiotherapy and 31% after chemotherapy). 44.3% complained of the inability of mouth opening. 30.8% reported a change in taste sensation, of which 12% had completely lost their sense of taste. 26.2% reported difficulty speaking and 12.6% reported difficulty eating. (Table 4)

2.8% of patients underwent tooth extraction after SCC treatment, but no osteoradionecrosis was reported in any patient.

**Table 4:** Prevalence of oral complication

Oral complication	Percent
Xerostomia	97.4%
Mucositis	46.2%
Inability to mouth opening	44.3%
Taste change	30.8%
Difficulty to speak	26.2%
Difficulty to swallow	12.6%

The incidence of treatment complications was not significantly related to age, sex, alcohol consumption and financial status. (p > 0.05)

Mucositis had a significant relationship with education level; (p = 0.024) the prevalence of mucositis was less in patients with higher education.

The level of education was significantly related to taste changes (p = 0.04), and with the increasing level of education, the taste change was less.

The incidence of mucositis was significantly associated with smoking (p = 0.04), and the incidence of mucositis was higher in smokers.

The recurrence rate was not significantly related to age, sex, financial status, education, alcohol and smoking.

## Discussion

As mentioned earlier, SCC is the eighth most common cancer globally, affecting many people each year [8]. SCC has various treatments such as surgery, radiotherapy, chemotherapy, etc. Each of these treatments cause complications for the patients that affect the quality of their life.

The most common complication following SCC treatment in our patients was xerostomia (97.4%). This finding was similar to the studies of Taoran Cui [9], Kristine Loken Westgaard [10] and Jenson AD [11], but in Rajesh V.Lalla’s study [12], the most common complication was reported as mucositis. The reason for this difference can be attributed to the time of research and examination of patients so that in Rajesh’s study, patients were examined six months after treatment (short-term complications). Still, in this study, a long time had elapsed since treatment.

The cause of xerostomia in these patients may be due to the removal of part of the salivary glands during surgery or because of radiotherapy of the face; the most common site of oral SCC is the tongue, so during radiotherapy, the salivary glands of the tongue and other salivary glands, including the parotid, are destroyed by radiation. Xerostomia is a complication that persists in patients for a long time. In a 2017 study by Primoz Strogan [13] et al., Radiotherapy was identified as the leading cause of xerostomia in patients with head and neck cancer; Of course, other factors such as age, sex, alcohol drinking and smoking can also play a role as contributing factors. Another cause of xerostomia was the significant decrease in saliva flow as well as PH and buffering capacity after radiotherapy [14]. In 2019 Baudelet found that xerostomia was the most common complication of long-term follow-up (3 to 8 years) [15].

The second most common complication among our patients was mucositis (46.2%). It is similar to Y Sroussi [16] and Rajesh.VLala [12] studies, this complication is acute due to damage to oral tissues after radiotherapy or chemotherapy. This complication is associated with a lot of pain and burning, which greatly affects the quality of life of patients. The Prevalence of mucositis is more after radiotherapy than chemotherapy. If radiotherapy and chemotherapy are performed simultaneously, the severity of the mucositis will increase. However, the severity of mucositis depends on the dose of radiotherapy and the type of tissues. Mucositis develops in people who receive radiotherapy, which greatly affects their quality of life and increases their need for psychological treatment [17, 18].

The third complication was difficulty in mouth opening (44.3%). This complication can occur following surgical treatment or radiotherapy. If the anatomical structures and muscles are removed during the surgical treatment, it can cause limitations in mouth opening. During radiotherapy treatment, the jaw muscles may develop fibrosis, which restricts the opening of the mouth. Similar to our result, Sook.Y .Loh [19] found that trismus is one of the most common complications of cancer treatment, which can result from surgical removal of anatomical structures and muscle fibrosis following radiotherapy. In another study conducted in 2017 by Sarah J. Bander, the prevalence of trismus among 730 patients with SCC was estimated at 23.6% [20]. In another study, the rate of mouth opening was examined in 671 patients, and in a quarter of patients, the rate of mouth opening was less than 33 mm [21].

In the present study, evaluating the causes or risk factors affecting the incidence of complications of cancer treatments, only mucositis had an inverse relationship with patients’ level of education (p = 0.024). The number of people with mucositis in patients with a bachelor’s degree or higher was significantly lower than other two groups, due to the fact that people with a higher level of education paid more attention to regular follow-ups and Frequent visits after treatment and recieved more supportive treatments that reduce mouth irritation. Also, these people have more precise oral hygiene, which makes them have a lower plaque index, which prevents inflammation. To date, no study has been conducted on this subject that we can compare our results with them.

Also, in people with a bachelor’s degree or higher, the rate of losing sense of taste was lower than in other groups. This case, similar to oral mucositis, can be attributed to the shorter duration of complications of cancer treatment due to the follow-up and use of existing treatments. No study has been done in this regard to compare the results.

Based on our findings, the incidence of mucositis was higher in smokers. (p = 0.04) due to the damage that smoking causes to the tissues, smokers are more prone to tissue damage. Bo-Young Hong reached a similar conclusion, and in his study, the severity of mucositis due to chemotherapy was significantly associated with smoking [22]. Another study by Beata Szeszko concluded that the severity of mucositis was higher in smokers, so 13.5% of them needed to be hospitalized to alleviate mucositis [23].

Lack of study time as well as small sample size, were the limitations of this study. Prevalence of Covid 19 disease and several months of quarantine and non-referral of patients, lack of cooperation of the families of the deceased, and lack of full response from other patients are other limitations of this study. It is suggested that in future studies in this field, more people and more valid questionnaires must be studied to assess the risk factors and quality of life of patients seeking treatment to achieve more results and more certainty.

## Conclusion

SCC treatments include surgery, radiotherapy and chemotherapy, which cause complications that have a significant impact on patients’ quality of life. The most common of these complications were xerostomia (97.4%), mucositis (46.2%) and limited mouth opening (44.3%), respectively. Mucositis had a significant relationship with the level of education, and the higher the education, the lower the percentage of mucositis reported. Increasing the level of education also led to a lower incidence of decreased taste in patients. In addition, the incidence of mucositis was significantly higher in smokers.

Frequent and regular follow-ups and supportive therapies will reduce these complications and improve patients’ quality of life.

## Data Availability

The datasets during the current study are not publicly available due to confidentiality of the patients’ data, but they will be available upon editorial reasonable request. Data will be available on request through the corresponding authors.

## References

[1] Shin Y (2019). Association of periodontitis with oral cancer: a case-control study. J Dent Res.

[2] Kowalski LP et al. *Survival trends of patients with oral and oropharyngeal cancer treated at a cancer center in São Paulo, Brazil*. Clinics, 2020. 75. 10.6061/clinics/2020/e1507 PMC7134554 32294669

[3] Printz C (2013). Highlights from the ASCO annual meeting. Cancer.

[4] Wong YF (2015). Treatment outcome for head and neck squamous cell carcinoma in a developing country: university Malaya medical centre, Malaysia from 2003–2010. Asian Pac J Cancer Prev.

[5] Liu F, Chen F, Huang J (2017). Prospective study on factors affecting the prognosis of oral cancer in a chinese population. Oncotarget.

[6] de Melo GM, Ribeiro KC, Kowalski LP (2001). Risk factors for postoperative complications in oral cancer and their prognostic implications. Arch Otolaryngol Head Neck Surg.

[7] Colares N (2019). Smoking history decreases survival in patients with squamous cell carcinoma of the mouth: a retrospective study with 15 years of follow-up. Asian Pac J cancer prevention: APJCP.

[8] Scully C, Bagan J (2009). Oral squamous cell carcinoma overview. Oral Oncol.

[9] Cui T (2019). Correlation between plan quality improvements and reduced acute dysphagia and xerostomia in the definitive treatment of oropharyngeal squamous cell carcinoma. Head Neck.

[10] Westgaard KL (2021). Oral and ocular late effects in head and neck cancer patients treated with radiotherapy. Sci Rep.

[11] Jensen A, Langer C. Late toxicity following primary conservative treatment: Dysphagia and xerostomia. HNO; 2020. 10.1007/s00106-020-00961-7 33180145

[12] Lalla RV (2017). Oral complications at 6 months after radiation therapy for head and neck cancer. Oral Dis.

[13] Strojan P (2017). Treatment of late sequelae after radiotherapy for head and neck cancer. Cancer Treat Rev.

[14] Arrifin A (2018). The effect of radiotherapy for treatment of head and neck cancer on oral flora and saliva. Oral Health Prev Dent.

[15] Baudelet M (2019). Very late xerostomia, dysphagia, and neck fibrosis after head and neck radiotherapy. Head Neck.

[16] Sroussi HY (2017). Common oral complications of head and neck cancer radiation therapy: mucositis, infections, saliva change, fibrosis, sensory dysfunctions, dental caries, periodontal disease, and osteoradionecrosis. Cancer Med.

[17] Maria OM, Eliopoulos N, Muanza T (2017). Radiation-induced oral mucositis. Front Oncol.

[18] Barma MD (2021). Quality of life among head and neck cancer treated patients in South India: a cross-sectional study. J Oral Biology Craniofac Res.

[19] Loh SY, Mcleod RW, Elhassan HA. Trismus following different treatment modalities for head and neck cancer: a systematic review of subjective measures. Volume 274. European Archives of Oto-Rhino-Laryngology; 2017. pp. 2695–707. 7. 10.1007/s00405-017-4519-6 PMC5486547 28343337

[20] van der Geer SJ (2019). Prevalence and prediction of trismus in patients with head and neck cancer: a cross-sectional study. Head Neck.

[21] Van Der Geer SJ (2019). Criterion for trismus in head and neck cancer patients: a verification study. Support Care Cancer.

[22] Hong B-Y (2019). Chemotherapy-induced oral mucositis is associated with detrimental bacterial dysbiosis. Microbiome.

[23] Szeszko B (2015). Smoking during radiotherapy for head and neck cancer and acute mucosal reaction. Rep Practical Oncol Radiotherapy.

